# SGLT1 Knockdown Attenuates Cardiac Fibroblast Activation in Diabetic Cardiac Fibrosis

**DOI:** 10.3389/fphar.2021.700366

**Published:** 2021-06-24

**Authors:** Hui Lin, Le Guan, Liping Meng, Hiroyasu Uzui, Hangyuan Guo

**Affiliations:** ^1^ Department of Cardiology, Shaoxing People’s Hospital, Shaoxing Hospital, Zhejiang University School of Medicine, Shaoxing, China; ^2^ Department of Radiology, Shaoxing People’s Hospital, Shaoxing Hospital, Zhejiang University School of Medicine, Shaoxing, China; ^3^ Department of Cardiovascular Medicine, Faculty of Medical Sciences, University of Fukui, Fukui, Japan; ^4^ College of Medicine, Shaoxing University, Shaoxing, China

**Keywords:** cardiac fibroblasts, sodium–glucose cotransporter, high glucose, diabetic cardiomyopathy, mitogen-activated protein kinase

## Abstract

**Background:** Cardiac fibroblast (CF) activation is a hallmark feature of cardiac fibrosis in diabetic cardiomyopathy (DCM). Inhibition of the sodium-dependent glucose transporter 1 (SGLT1) attenuates cardiomyocyte apoptosis and delays the development of DCM. However, the role of SGLT1 in CF activation remains unclear.

**Methods:** A rat model of DCM was established and treated with si‐SGLT1 to examine cardiac fibrosis. In addition, *in vitro* experiments were conducted to verify the regulatory role of SGLT1 in proliferation and collagen secretion in high-glucose– (HG–) treated CFs.

**Results:** SGLT1 was found to be upregulated in diabetic cardiac tissues and HG-induced CFs. HG stimulation resulted in increased proliferation and migration, increased the expression of transforming growth factor-β1 and collagen I and collagen III, and increased phosphorylation of p38 mitogen-activated protein kinase and extracellular signal-regulated kinase (ERK) 1/2. These trends in HG-treated CFs were significantly reversed by si-SGLT1. Moreover, the overexpression of SGLT1 promoted CF proliferation and collagen synthesis and increased phosphorylation of p38 mitogen-activated protein kinase and ERK1/2. SGLT1 silencing significantly alleviated cardiac fibrosis, but had no effect on cardiac hypertrophy in diabetic hearts.

**Conclusion:** These findings provide new information on the role of SGLT1 in CF activation, suggesting a novel therapeutic strategy for the treatment of DCM fibrosis.

## Introduction

Diabetic cardiomyopathy (DCM) is a myocardial disease that is specific to patients with diabetes and is independent of various types of heart diseases, including hypertension, coronary, and valvular (Bugger and Abel, 2014; Seferovic and Paulus, 2015). Cardiac fibrosis caused by abnormal glucose metabolism and microangiopathy are the main pathological features of DCM, leading to impairment of cardiac function and eventual progression to heart failure (Wang et al., 2021). The activation of cardiac fibroblasts (CFs) and degeneration of cardiomyocytes provide the biological basis for cardiac remodeling and the pathophysiological basis of DCM formation (Zhang et al., 2020). CFs switched from a resting type to an activated type increasing their proliferation and migration capacity and began to secrete large amounts of extracellular matrix, causing fibrosis of the heart (Frangogiannis, 2021). The development of fibrosis-targeting therapies for patients with DCM will help to further understand the functional pluralism of CFs and dissect the molecular basis for fibrotic remodeling.

Sodium–glucose cotransporter (SGLT) belongs to the solute carrier five gene family, which transports glucose against a concentration gradient in an energy-consuming manner and plays an important role in the active transport of glucose (Wood and Trayhurn, 2003; Sano et al., 2020). Sodium–glucose cotransporter 1 (SGLT1) is expressed in various human tissues and organs, including the intestine, lung, heart, skeletal muscle, and kidney (Gyimesi et al., 2020). SGLT1 is essential for the quick absorption of glucose and galactose in the intestine, and increases in SGLT1 protein expression cause interstitial fibrosis and cardiac remodeling in mice (Ramratnam et al., 2014). SGLT1 expression is also elevated in hypertrophic cardiomyopathy, ischemic cardiomyopathy, and DCM in humans (Song et al., 2016). Selective inhibition of SGLT1 expression has a protective effect against myocardial-infarction-induced ischemic cardiomyopathy (Sawa et al., 2020). In addition, Hirose et al. (2018) demonstrated that SGLT1 knockout effectively alleviated pressure-overload–induced cardiomyopathy, suggesting that SGLT1 inhibitors have an active effect on hypertrophic cardiomyopathy. More importantly, our previous study found that SGLT1 inhibition could attenuate apoptosis and relieve myocardial fibrosis, thus suppressing DCM development by regulating the JNK/p38 signaling pathway (Lin et al., 2021). However, in the abovementioned study, we only investigated the role of SGLT1 in cardiomyocytes and rat H9C2 cells. It would be more appropriate to study the role of SGLT1 in the activation of CFs during the development of DCM.

Our previous study found that high-glucose (HG) levels promote SGLT1 and matrix metalloproteinase 2 expression in CFs (Meng et al., 2018), but whether HG levels promote cardiac fibrosis by inducing CF activation and whether SGLT1 is involved in HG-induced CF activation have not been reported. Thus, arrays of experiments were performed in this study to determine the role of SGLT1 in CF activation during DCM. Moreover, we tried to characterize the role of the p38 and ERK1/2 signaling pathways in the regulatory mechanism of SGLT1 expression for CF activation.

## Materials and Methods

### Ethics Statement

All animal procedures were conducted in accordance with the National Institutes of Health Guide for the Care and Use of Laboratory Animals and were approved by the Medicine Animal Welfare Committee of Shaoxing People’s Hospital.

### Culture of Rat Primary CFs

Primary rat CFs were isolated from the ventricles of neonatal male Sprague Dawley rats (2–3 days old) using enzyme digestion. The tissue was cut into 1 mm cube pieces and digested with trypsin/EDTA (Gibco, NY, United States) and collagenase II (Sigma, United States) at 37°C. The mixture (collagenase and trypsin, 100: 1) was placed in a shaker at 37°C for 20 min, and the supernatant was collected and combined with DMEM containing 10% FBS, and this process was repeated until the tissue was fully digested. Cardiomyocytes were separated from CFs using centrifugation at a low speed (300 g), and the supernatant containing CFs was collected. The isolated CFs were cultured in DMEM supplemented with 10% FBS and 1% penicillin–streptomycin at 37°C in a humidified incubator with 5% CO_2_. Cells were divided into four groups: 1) a control group, in which CFs were incubated with DMEM containing 5.5 mmol/L normal glucose for 48 h; 2) an HG group, in which CFs were incubated with DMEM containing 33 mmol/L glucose (HG) for 48 h; 3) an HG + si-NC group, in which CFs were transfected with si-NC and cultured under HG conditions for 48 h; and 4) an HG + si-SGLT1 group, in which CFs were transfected with si-SGLT1 and cultured under HG conditions for 48 h.

### Small Interference RNA Transfection

To knockdown the expression of SGLT1 in CFs, small interfering RNAs against the SGLT1 gene (si-SGLT1) and the siRNA negative control (si-NC) were synthesized at Guangzhou RiboBio Co., Ltd. Briefly, CFs grown to 70–80% confluence were incubated with Lipofectamine 3,000 transfection reagent (Invitrogen, Waltham, MA, United States) loaded with siRNAs for 48 h. Transfection efficiency was evaluated using RT-qPCR analysis.

### Cell Counting Kit-8 Assay

CFs were cultured in 96-well plates at a density of 1×10^4^ cells/well. A 10 µl aliquot of the Cell Counting Kit-8 (MCE, Shanghai, China) solution was added to each well, and the plates were incubated at 37°C for 1 h. Absorbance was measured using a microplate reader (Molecular Devices, CA) at 450 nm.

### Wound Scratch Assay

Cells were grown to 90% confluence in 6-well plates in DMEM supplemented with 10% FBS, and the medium was replaced with serum-free DMEM to starve cells for 24 h. Wounds were made with a sterile 200 μl pipette tip by drawing a line through the plated cells perpendicular to the abovementioned line. CFs were transfected with si-NC or si-SGLT1 and exposed to HG conditions for 24 h. Images were acquired using a Leica microscope (DM 2000, Leica, Wetzlar, Germany).

### Western Blotting

Protein samples from CFs and cardiac tissues were extracted using the RIPA buffer, and equal amounts of proteins from each group were separated by 12% sodium dodecyl sulfate-polyacrylamide gel electrophoresis. Then, the separated proteins were transferred to polyvinylidene fluoride membranes and blocked using 5% nonfat milk for 1 h at room temperature. Subsequently, the membranes were incubated with primary antibodies at 4°C overnight, followed by incubation with horseradish peroxidase– (HRP–) conjugated goat anti-rabbit secondary antibody (1:5,000, Santa Cruz) for 1 h at room temperature. The following primary antibodies were purchased from Abcam and used at a 1:1,000 dilution: β-actin (ab5694), SGLT1 (ab14686), collagen I (ab34710), and collagen III (ab6310). The following antibodies were purchased from Cell Signaling Technology and used at a 1:1,000 dilution: ERK1/2 (cat. 4695), phospho-ERK1/2 (cat. 4376), p38 (cat. 8690), and phospho-p38 (cat. 4511).

### RNA Analysis

TRIzol reagent (Invitrogen, Carlsbad, CA, United States) was used to extract RNA from cardiac tissues, and cDNA was synthesized using a PrimeScript Reverse Transcription Reagent Kit (Takara, Otsu, Japan). RT-PCR was performed using a SYBR Premix Ex Taq Kit (Takara). The following primer sequences were used: SGLT1 forward, 5′-GGA​CAG​TAG​CAC​CTT​GAG​C-3′; reverse, 5′-CCA​ACA​GTC​CCA​CGA​TTA​G-3′; β-actin forward, 5′-CCA​GAT​CAT​GTT​TGA​GAC​CT-3′; and reverse, 5′-TCT​CTT​GCT​CGA​AGT​CTA​GG-3′. All reactions were performed in triplicate.

### Animal Experiments

Sprague Dawley rats were obtained from the Nanjing Biomedical Research Institute of Nanjing University (China). A total of 24 6-week-old male SD rats were assigned to four groups (control, STZ, si-NC, and si-SGLT1 groups) using the random number method, with six rats in each group. A 12-h light–dark cycle was used, and the rats were provided *ad libitum* access to food and water. After acclimation for 1 week, rats in the diabetes groups were fed a high-fat diet (fat provided 60% of total calories, Research Diet D12492) for 4 weeks and then intraperitoneally injected with 60 mg/kg STZ (Sigma) dissolved in a citrate buffer (pH 4.5), whereas the control group received normal chow. We performed intraperitoneal glucose tolerance tests (IPGTTs) to identify the insulin-resistant rats, and fasting blood glucose (FBG) levels were measured seven days after injection. Body weights were recorded. Successful induction of diabetes was defined by an FBG value higher than 16.7 mmol (Feng et al., 2019). After successful establishment of the rat model with DCM, the rats in the si-NC and si-SGLT1 groups were injected with 5 μl of siRNA or 5 μl of si‐SGLT1 (200 nmol/500 g) in PBS, once a week. All rats were sacrificed after 16 weeks of feeding. The left ventricular tissues were removed and cut into pieces for histomorphological analysis.

### ELISA

After the rats were fasted overnight, blood samples were obtained from the postcaval vein and processed for plasma extraction within 1 h (centrifuged at 3,000 x g for 10 min at 4°C), and the plasma was stored at –80°C in polypropylene tubes for further analysis. The expression levels of collagen I, collagen III, and transforming growth factor-β1 (TGF-β1) in CFs and rat serum were detected using the rat collagen I Type I ELISA Kit (abx052369, Abbexa, United Kingdom), rat collagen type III ELISA Kit (abx573727, Abbexa), and TGF-β1 ELISA Kit (PT878, Beyotime, Jiangsu, China), respectively, following the instructions of the manufacturer.

### Histology

Tissues from rats were fixed using 10% buffered formalin, dehydrated, embedded in paraffin, and sectioned into 5 μm-thick sections. Hematoxylin and eosin (HE) staining was used to assess cardiac injury, whereas Masson’s trichrome staining was used to detect collagen fibers, and the slides were observed under an optical microscope. For immunohistochemistry, sections were stained with a primary antibody against SGLT1 (1:200, Abcam) and then stained with a secondary antibody. After washing with PBS, the slides were incubated with 3,3′-diaminobenzidine. The detailed procedure has been described previously (Lin et al., 2019). Semiquantitative analysis was performed using image analysis software (Image-Pro Plus, Media Cybernetics).

### Wheat Germ Agglutinin Staining

Slides were stained with Alexa Fluor 488–conjugated wheat germ agglutinin WGA (Sigma). In brief, slides were dewaxed, rehydrated, and blocked with 3% BSA for 20 min. The slides were then incubated in WGA solubilized in PBS for 30 min at room temperature in the dark. After washing with PBS, the sections were stained with DAPI (Invitrogen) for 5 min and images were acquired using a Nikon Eclipse Ti‐U fluorescence microscope (Minato‐ku, Tokyo, Japan). The cardiomyocyte size was determined by dividing the total area by the number of cardiomyocytes using Image J software (NIH, Bethesda, MD, Unites States).

### Statistical Analysis

The experimental data are expressed as the mean ± standard deviation. All statistical analyses were performed using GraphPad Prism 8.0 software (GraphPad Software, San Diego, CA, United States). Data are presented as the mean ± standard deviation. The *t*-test was used to perform comparisons between the two different groups. One-way analysis of variance was used to compare multiple groups. Statistical significance was set at *p* < 0.05.

## Results

### SGLT1 Is Upregulated in Diabetic Cardiac Tissues and High-Glucose–Induced Cardiac Fibroblasts

**FIGURE 1:**
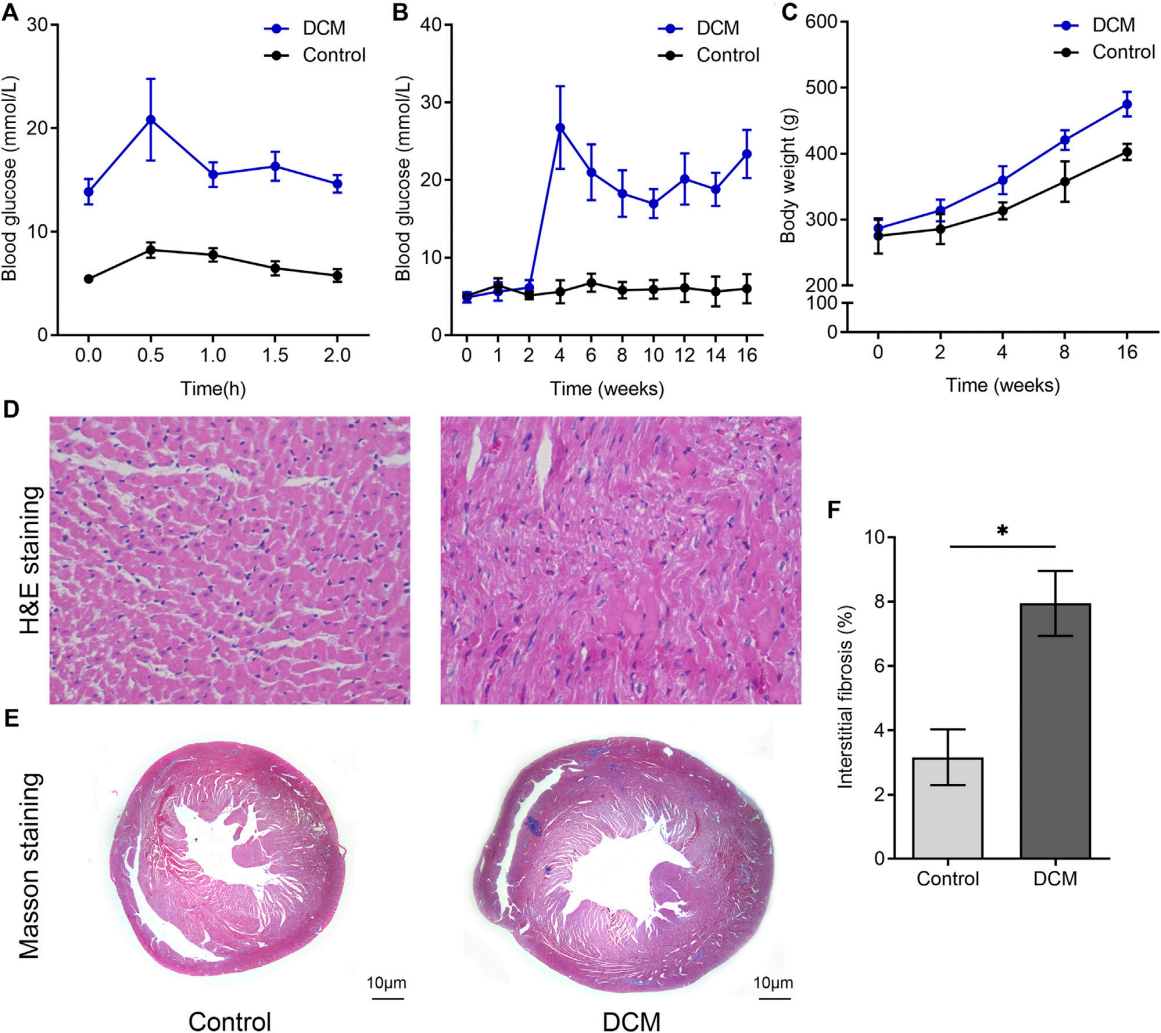
Changes of blood glucose level and histopathology in DCM rats. SD rats were fed a high-fat diet (HFD) for 4 weeks before streptozotocin (STZ) was injected intraperitoneally at a dose of 60 mg/kg, and rats were fed for another 12 weeks. **(A)** Intraperitoneal glucose tolerance tests (IPGTTs) were used to identify insulin resistance in the two groups. **(B)** The FBG levels of the two groups measured at indicated times. **(C)** The body weight of the two groups was monitored at the indicated time. The paraffin sections of rat left ventricular tissues after **(D)** hematoxylin and eosin (H&E) staining and **(E)** Masson’s trichrome staining. **(F)** Quantitative analysis of interstitial Masson’s trichrome staining (*n* = 6 per group).

The results of IPGTT revealed that blood glucose levels peaked half an hour after intraperitoneal injection, slowly decreased thereafter, and remained at 6–8 mmol/L in the control group throughout the entire process. In contrast, the blood glucose levels of diabetic rats demonstrated evident hyperglycemia throughout the entire process (Figure 1A). Figure 1B shows that rats in the control group had a relatively stable FBG level, whereas the FBG level of the DCM group increased significantly after injection with STZ in the fourth week (*p* < 0.05). The body weight of the DCM group was higher than that of the control group (Figure 1C). HE and Masson staining were performed to examine the changes in cardiac pathology (Figures 1D,E). Increased interstitial fibrotic areas were observed in the DCM group compared to the NC group (Figure 1F).

**FIGURE 2:**
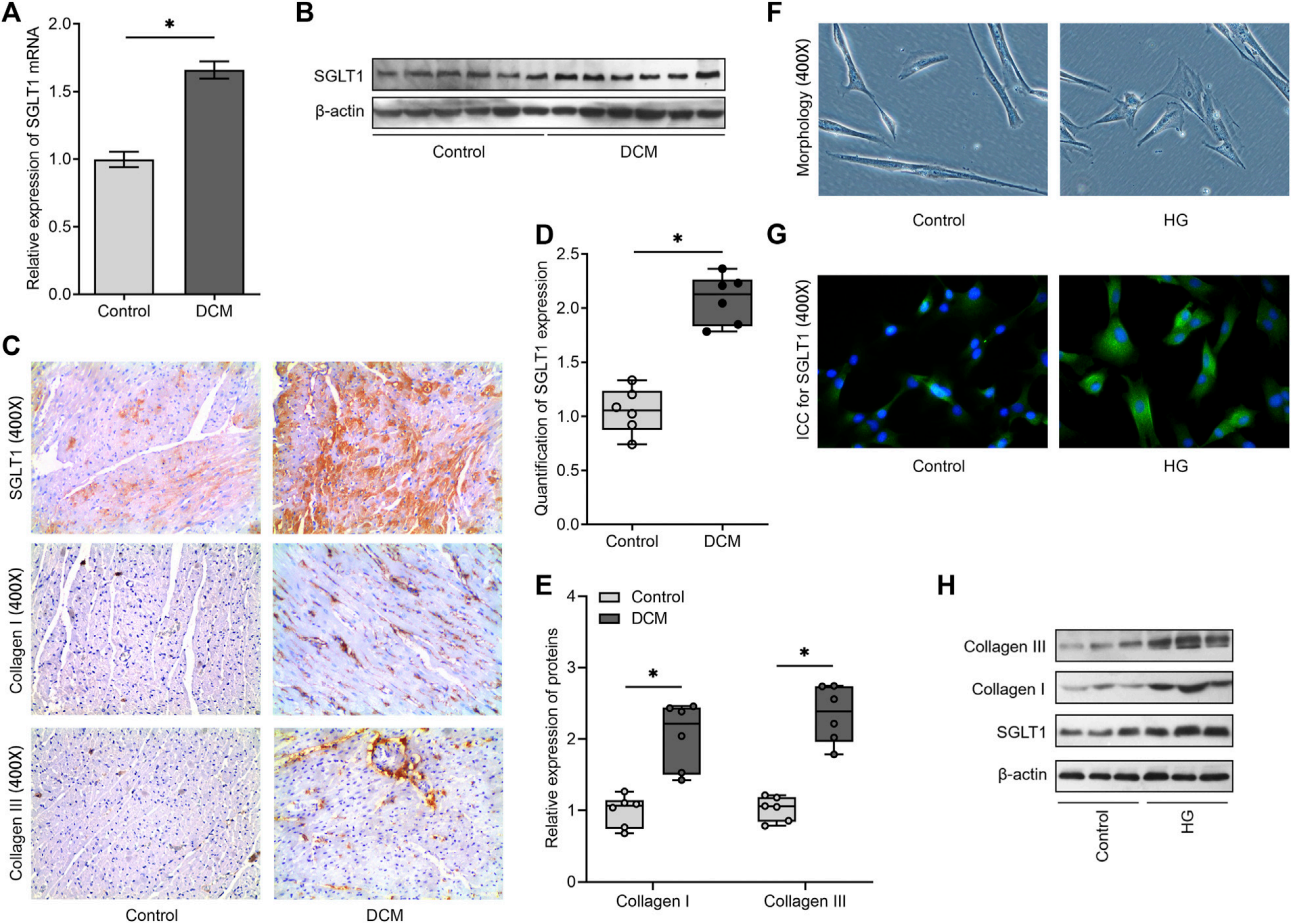
SGLT1 is downregulated in the experimental diabetic cardiac fibrosis model and high-glucose–induced CFs. **(A)** The mRNA level of SGLT1 was detected using RT‐qPCR (*n* = 6). **(B)** The protein level of SGLT1 was analyzed using western blot analysis (*n* = 6). **(C)** The protein levels of SGLT1, collagen I, and collagen III were evaluated using immunohistochemical staining. Relative expression of SGLT1 **(D)** and collagen I and collagen III **(E)** was found using semiquantitative analysis based on immunohistochemistry. **(F)** The morphology of CFs with or without high-glucose stimulation was imaged under an optical microscope. **(G)** The protein level of SGLT1 was analyzed by immunofluorescent staining in high-glucose–induced CFs. **(H)** The protein levels of SGLT1, collagen I, and collagen III were analyzed using western blot analysis in high-glucose–induced CFs (*n* = 3).

We then validated that SGLT1 mRNA and protein levels were both upregulated in the DCM group compared to those in the control group (Figures 2A,B). Moreover, using immunohistochemistry, we found that CFs in diabetic rats expressed higher SGLT1 levels than those in normal rats (Figures 2C,D). In contrast to the control group, diabetic rats showed a significant increase in fibrosis-related proteins, including collagen I and collagen III expression (Figures 2C,E). *In vitro*, CFs changed from a long and thin shape to fusiform under HG conditions (Figure 2F), and the results of immunofluorescence revealed that the fluorescence intensity of SGLT1 was markedly increased in the HG medium (Figure 2G). Accordingly, western blotting analysis revealed that compared to the control group, the levels of SGLT1, collagen I, and collagen III proteins were significantly upregulated in the HG group (Figure 2H).

### Knockdown of SGLT1 Inhibits High-Glucose–Induced Cardiac Fibroblast Activation

**FIGURE 3:**
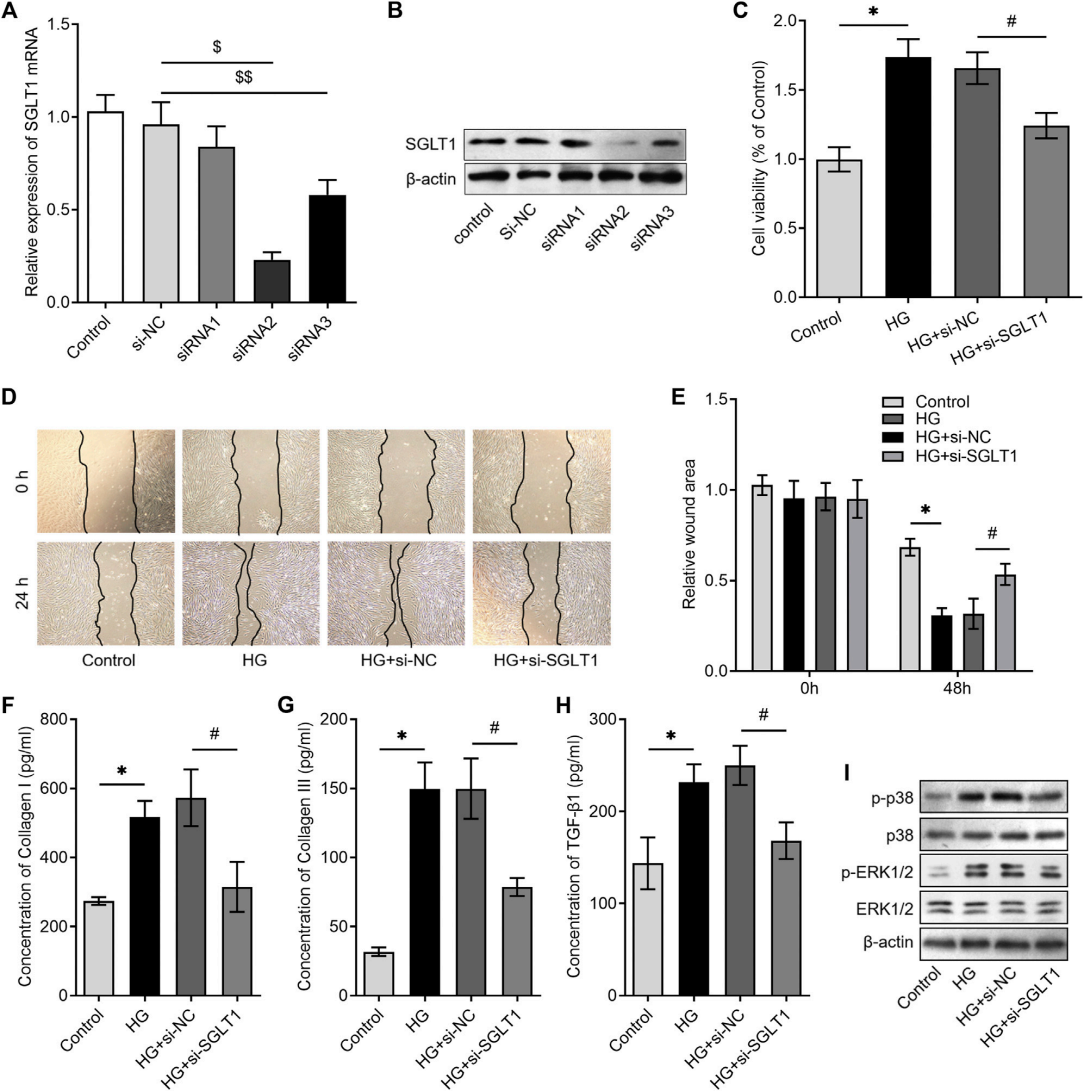
Knockdown of SGLT1 inhibited high-glucose–induced CF activation. CFs were transfected with SGLT1 siRNAs, and SGLT1 mRNA and protein levels were detected using RT‐qPCR **(A)** and western blotting, respectively **(B)**. CCK-8 assay was used to detect the proliferation of CFs under high-glucose condition with or without SGLT1 inhibition. **(D, E)** The representative images of the wound-healing assay were obtained at 0 and 24 h after knockdown of SGLT1, and the migrative ability of CFs was compared. **(F–H)** ELISA was used to detect the levels of collagen-synthesis–related markers, including TGF-β1, collagen I, and collagen III in the cell supernatant (*n* = 6). **(I)** Western blotting analysis was performed to investigate the phosphorylation levels of p38 mitogen-activated protein kinase (MAPK) and extracellular signal-regulated kinase (ERK)1/2 in CFs under high-glucose condition with or without SGLT1 inhibition.

To investigate the role of SGLT1 in CF activation, we knocked down SGLT1 by transfecting CFs with specific siRNAs against SGLT1. The results of RT-qPCR (Figure 3A) and western blotting (Figure 3B) analysis validated that SGLT1 si-RNA2 exerted the highest knockdown efficiency and was, therefore, chosen to perform the subsequent assays. Subsequent characterization of the CFs showed that HG stimulation significantly increased cell viability (Figure 3C) and migration (Figures 3D,E). We found that CFs with SGLT1 inhibition had markedly reduced cell viability and migration compared with those in the HG + si-NC group (Figures 3C–E). Furthermore, ELISA revealed that HG stimulation caused high expression of TGF-β1, collagen I, and collagen III, indicating the synthesis of collagen in CFs, whereas the inhibition of SGLT1 effectively reversed this increase (Figures 3F–H). We further analyzed the potential involvement of the p38 mitogen-activated protein kinase (MAPK) and ERK1/2 signaling pathways in the regulatory role of SGLT1 in HG-mediated CF activation. As expected, HG significantly activated the phosphorylation of p38 MAPK and ERK1/2, whereas SGLT1 silencing reduced the effects of HG (Figure 3I). Therefore, these data indicate that SGLT1 regulates the function of CFs.

### Sodium–Glucose Cotransporter 1 Regulates p38 MAPK and ERK1/2 Signaling and Collagen Synthesis in Cardiac Fibroblasts

**FIGURE 4:**
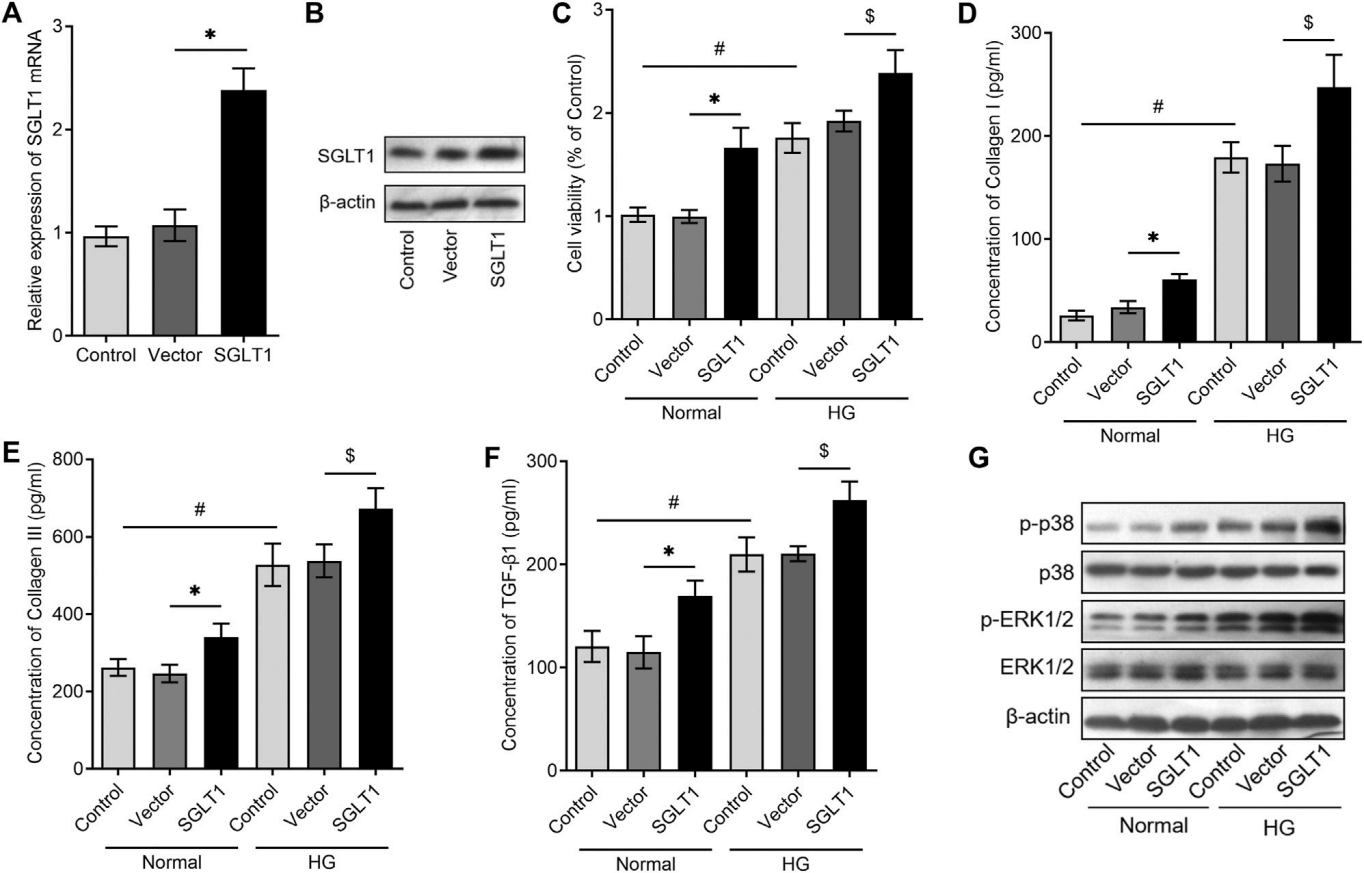
SGLT1 overexpression promotes collagen release *via* the p38 and ERK1/2 signaling pathway in CFs. CFs were transfected with an SGLT1 plasmid or vector, and SGLT1 expression levels were detected using RT‐qPCR **(A)** or western blotting analysis **(B)**. **(C)** After overexpression of SGLT1 in CFs, CCK-8 assay was used to detect the proliferation of CFs under normal or high-glucose condition (*n* = 6). **(D–F)** After overexpressing SGLT1 in CFs, the collagen-synthesis–related markers, including TGF-β1, collagen I, and collagen III, in the cell supernatant were measured using ELISA (*n* = 6). **(G)** After overexpressing SGLT1 in CFs, the protein levels of phosphorylated p38 and p-ERK1/2 were analyzed using western blotting in CFs both under normal or high-glucose condition (*n* = 3).

We further investigated the potential mechanism underlying the involvement of SGLT1 in the activation of CFs. SGLT1 overexpression was achieved by transfecting CFs with a plasmid containing the SGLT1 gene, and RT-qPCR and western blotting analysis revealed significantly higher SGLT1 levels in the SGLT1-transfected group when compared with the control group (Figures 4A,B). Interestingly, we found that the overexpression of SGLT1 significantly increased the proliferation of CFs under both normal and HG conditions (Figure 4C). In addition, the overexpression of SGLT1 effectively promoted the secretion of TGF-β1, collagen I, and collagen III in the cell supernatant, under both normal and HG conditions (Figures 4D–F). Furthermore, overexpression of SGLT1 significantly increased p38 and p-ERK1/2 levels under both normal and HG conditions (Figure 4G). These data suggest that SGLT1 might activate CFs by activating the p38 and p-ERK1/2 pathways.

### Sodium–Glucose Cotransporter 1 Inhibition Alleviates Fibrosis in the Diabetic Heart

**FIGURE 5:**
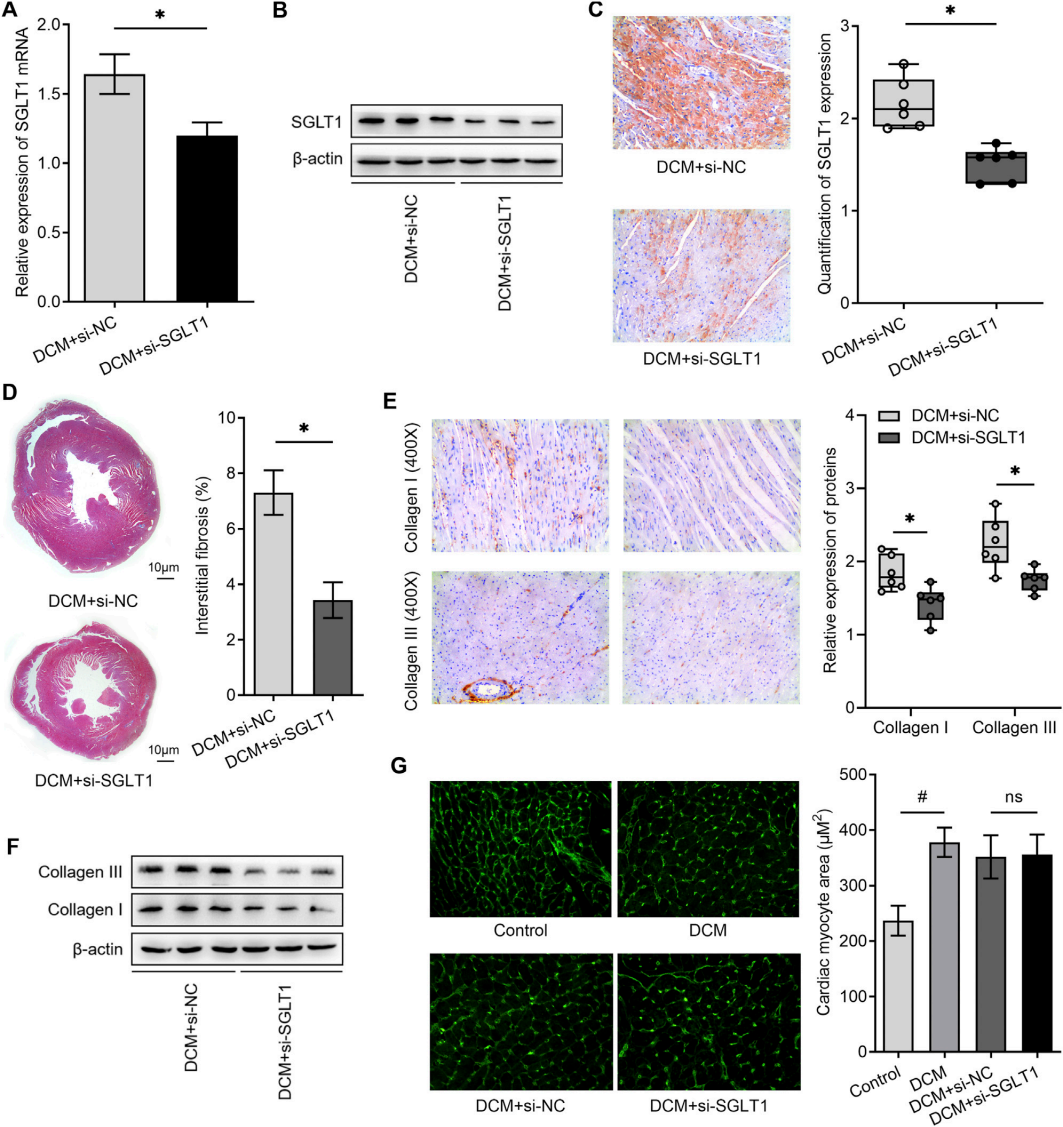
SGLT1 inhibition alleviated myocardial fibrosis and apoptosis in diabetic rat hearts (*n* = 6). **(A, B)** Knockdown of SGLT1 was achieved by continuous intravenous injection into the tail vein with specific SGLT1 siRNA in DCM rats, and the knockdown efficiency was identified by detecting SGLT1 expression in myocardial tissues using RT-qPCR and western blotting analysis. **(C)** SGLT1 protein levels in myocardial tissue were measured by immunohistochemistry staining. **(D)** Masson’s trichrome staining of myocardial tissue shows interstitial fibrosis in DCM rats treated with si-NC or si-SGLT1. **(E, F)** Relative protein levels of collagen I and collagen III in myocardial tissue were measured using immunohistochemical staining and western blotting analysis (*n* = 6). **(G)** Wheat germ agglutinin staining and quantitation of myocardial cell size in DCM rats treated with si-NC or si-SGLT1 (*n* = 6).

Since cardiac fibrosis and hypertrophy are important pathological structural features of DCM, we investigated whether SGLT1 regulates fibrosis and hypertrophy in diabetic hearts. We knocked down SGLT1 in rats with DCM by continuously administering si-SGLT1 *via* intravenous injection in the tail vein. As shown in Figures 5A,B, the expression of SGLT1 mRNA and protein in the heart was reduced in the DCM + si-SGLT1 group when compared with that in the DCM + si-NC group. Accordingly, the immunohistochemistry results indicated that CFs expressed low SGLT1 levels after SGLT1 inhibition (Figure 5C). Interestingly, compared with the DCM + si-NC group, a significant reduction in interstitial fibrosis was observed in the DCM + si-SGLT1 group (Figure 5D). The hallmarks of fibrosis and protein levels of collagen I and collagen III were reduced by SGLT1 knockdown (Figures 5E,F). However, SGLT1 knockdown had no significant effect on cardiac hypertrophy, as examined by WGA staining (Figure 5G). Collectively, our results indicated that the knockdown of SGLT1 reduced cardiac fibrosis but had no effect on cardiac hypertrophy in DCM.

## Discussion

Increasing attention has been placed on the utility of SGLT2 inhibitors because, in addition to controlling blood glucose levels, they have been shown to provide significant cardiovascular benefits in T2DM patients (Packer et al., 2021). Rather than SGLT2, recent findings have also emphasized the potential role of SGLT1 in the development of cardiovascular diseases. The myocardial expression of SGLT1 in humans is altered in various cardiovascular disease states. Compared with controls, left ventricular SGLT1 mRNA and protein expression was significantly upregulated in heart failure patients with DCM (Sayour et al., 2020). Individuals carrying loss-of-function mutations in the SGLT1 gene are estimated to have a lower risk of developing heart failure, driven by mitigation of postprandial hyperglycemic episodes (Seidelmann et al., 2018). In endothelial cells, angiotensin II upregulates SGLT1 expression to promote sustained oxidative stress, and inhibition of SGLT1 appears to be an attractive strategy to enhance protective endothelial function (Park et al., 2021). Apart from cardiomyocytes and endothelial cells in the heart, we first identified that SGLT1 was expressed in human CFs (Meng et al., 2018), and in the present study, the significant finding was that an increase in SGLT1 expression in rat hearts triggered the development of cardiac fibrosis through activation of CFs by upregulating the p38 MAPK and ERK1/2 signaling pathways.

Targeting SGLT1 has also been found to have cardioprotective effects in DCM. RNA-mediated inhibition of SGLT1 gene glycemic variability and cardiac damage were seen in type 2 diabetes mellitus mice *in vivo* (Sun et al., 2021). In cultured cardiomyocytes, SGLT1 knockdown restored cell proliferation, suppressed reactive oxygen species, and induced cytotoxicity (Chai et al., 2021). These data supported the notion that SGLT1 might serve as a target for myocardial injury in the diabetic heart. It is well known that CF activation plays an essential role during the development of cardiac fibrosis. However, the role of SGLT1 in CF activation remains unclear.

Several lines of experimental evidence suggest that SGLT1 silencing may attenuate cardiac fibrosis. Ramratnam et al. (2014) demonstrated that cardiac overexpression of SGLT1 increases collagen I gene expression and interstitial fibrosis in mouse hearts. Another study found that SGLT1 knockout downregulated CTGF and collagen I gene expression and interstitial fibrosis in pressure-overload–increased mouse hearts (Matsushita et al., 2018). Similar to these studies, we also found that knockdown of SGLT in diabetic hearts suppressed the synthesis of TGF-β1, collagen I, and collagen III. Furthermore, in cultured CFs, we found that SGLT1 regulates cell proliferation and collagen synthesis, suggesting the role of SGLT1 in regulating CF activation. To the best of our knowledge, this study is the first to demonstrate that SGLT1 regulates the activation of CFs in DCM.

Our *in vitro* experiments showed that HG upregulated SGLT1 expression in CFs, which was accompanied by an increase in the abundance of p-p38 and p-ERK1/2. SGLT1 overexpression significantly induced the abundance of these proteins in CFs under both normal and HG conditions. TGF-β1 stimulation in CFs resulted in increased proliferation, increased collagen I and collagen III expression, and increased p38 and ERK1/2 phosphorylation (Xu et al., 2017), whereas inhibition of the activation of p38 kinase and ERK1/2 could effectively attenuate cardiac fibrosis (Tao et al., 2016). Activation of MAPKs participates in the upregulation of cerebral SGLT-1 expression (Yamazaki et al., 2018). Moreover, the relationship between the SGLT1 and MAPK signaling pathways in the heart has also been reported in our previous study (Lin et al., 2021). Based on the abovementioned results, we deduced that the increase in SGLT1 expression in the diabetic heart is involved in triggering CF proliferation and subsequent cardiac fibrosis.

Furthermore, we noticed that the study performed by Matsushita et al. suggested that SGLT1 knockout could prevent chronic pressure-overload–induced hypertrophic cardiomyopathy (Matsushita et al., 2018). However, in our study, we found that knockdown of SGLT1 had no effect on hyperglycemia-related hypertrophy in diabetic hearts. This discrepancy may be because of the differences in experimental animal models. We used SD rats to establish a DCM model, and Matsushita et al. (2018) used mice that underwent transverse aortic constriction surgery. The other significant difference is that we only knocked down SGLT1 in rats using specific siRNA, rather than using gene knockout technology. A previous study suggested that SGLT1-deficient mice need to consume a glucose–galactose-free diet because they show symptoms of glucose–galactose malabsorption syndrome (Gorboulev et al., 2012). Therefore, SGLT1 knockout may not be appropriate in DCM. More studies are needed to investigate the exact role of SGLT1 in cardiac hypertrophy.

In summary, our study evaluated the changes in the expression of SGLT1 in the progression of diabetic cardiac fibrosis and identified a significant increase in SGLT1 expression in the diabetic heart. SGLT1 is involved in cardiac fibrosis via the p38 and ERK1/2 signaling pathways. Our findings suggest that SGLT1 is a potential therapeutic target for the prevention of diabetic cardiac fibrosis.

## Data Availability

The raw data supporting the conclusions of this article will be made available by the authors, without undue reservation.

## Abbreviations

DCM, diabetic cardiomyopathy; CFs, cardiac fibroblasts; SGLT1, sodium–glucose cotransporter; FBG, fasting blood glucose; IPGTTs, intraperitoneal glucose tolerance tests; HG, high glucose; MAPK, mitogen-activated protein kinase; ERK, extracellular signal-regulated kinase; TGF-β1, transforming growth factor-β1.
